# Recent advances in applications of multimodal ultrasound-guided photoacoustic imaging technology

**DOI:** 10.1186/s42492-020-00061-x

**Published:** 2020-10-21

**Authors:** Shanshan Wang, Yunfeng Zhao, Ye Xu

**Affiliations:** 1 VisualSonics Business Department, FUJIFILM (China) Investment Co. Ltd., Beijing, 100026 China; 2 VisualSonics Business Department, FUJIFILM (China) Investment Co. Ltd., Shanghai, 200120 China

**Keywords:** Photoacoustic/ultrasound imaging, The second near-infrared photoacoustic, Photothermal therapy, Photodynamic therapy, Multi-mode imaging

## Abstract

Photoacoustic imaging (PAI) is often performed simultaneously with ultrasound imaging and can provide functional and cellular information regarding the tissues in the anatomical markers of the imaging. This paper describes in detail the basic principles of photoacoustic/ultrasound (PA/US) imaging and its application in recent years. It includes near-infrared-region PA, photothermal, photodynamic, and multimode imaging techniques. Particular attention is given to the relationship between PAI and ultrasonic imaging; the latest high-frequency PA/US imaging of small animals, which involves not only B-mode, but also color Doppler mode, power Doppler mode, and nonlinear imaging mode; the ultrasonic model combined with PAI, including the formation of multimodal imaging; the preclinical imaging methods; and the most effective detection methods for clinical research for the future.

## Photoacoustic/ultrasound imaging

**Fig. 1:**
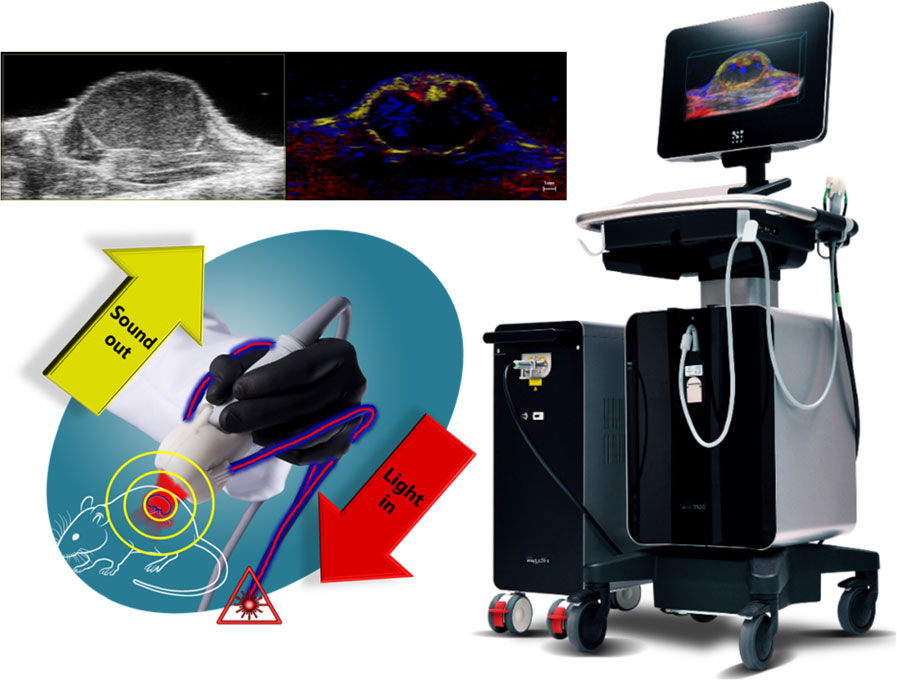
Schematic of PA/US multimode imaging system

**Fig. 2:**
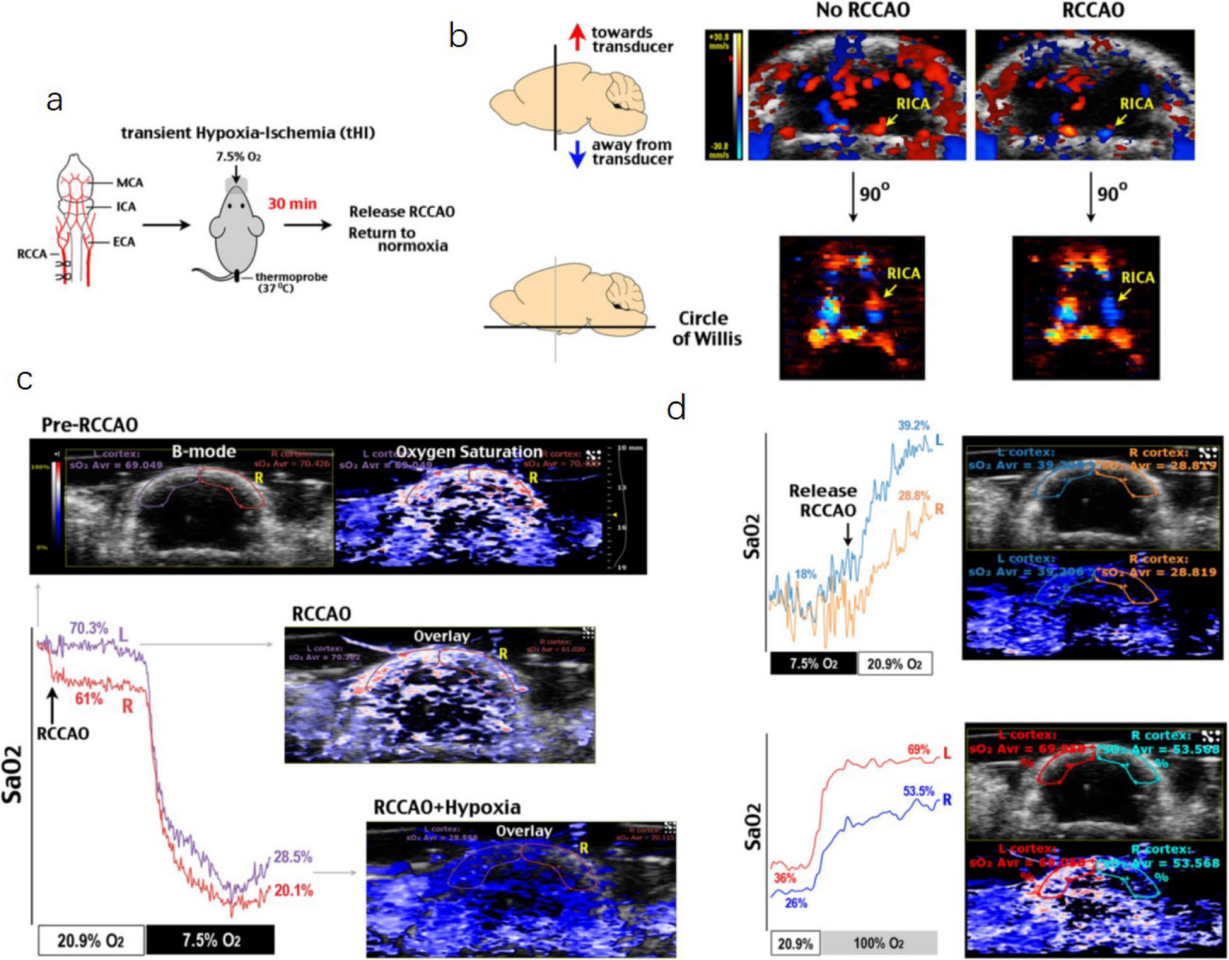
tHI caused prolonged deficits in cortical oxygenation. Doppler flow imaging and 3D reconstruction showed reduced perfusion in the ipsilateral hemisphere and the reversal of blood flow in the RICA in the Circle of Willis after RCCAO. Co-registered anatomical image (B-mode micro-US) and StO_2_ (StO_2_ in a red-white-blue color-map by PAI) showed bilateral difference in StO_2_ between the left (L, contralateral) and right (R, ipsilateral) cerebral cortex of an adult C57BL/6 mouse after RCCAO in normoxic (20.9% O_2_) or hypoxic (7.5% O_2_) conditions [16]

**Fig. 3:**
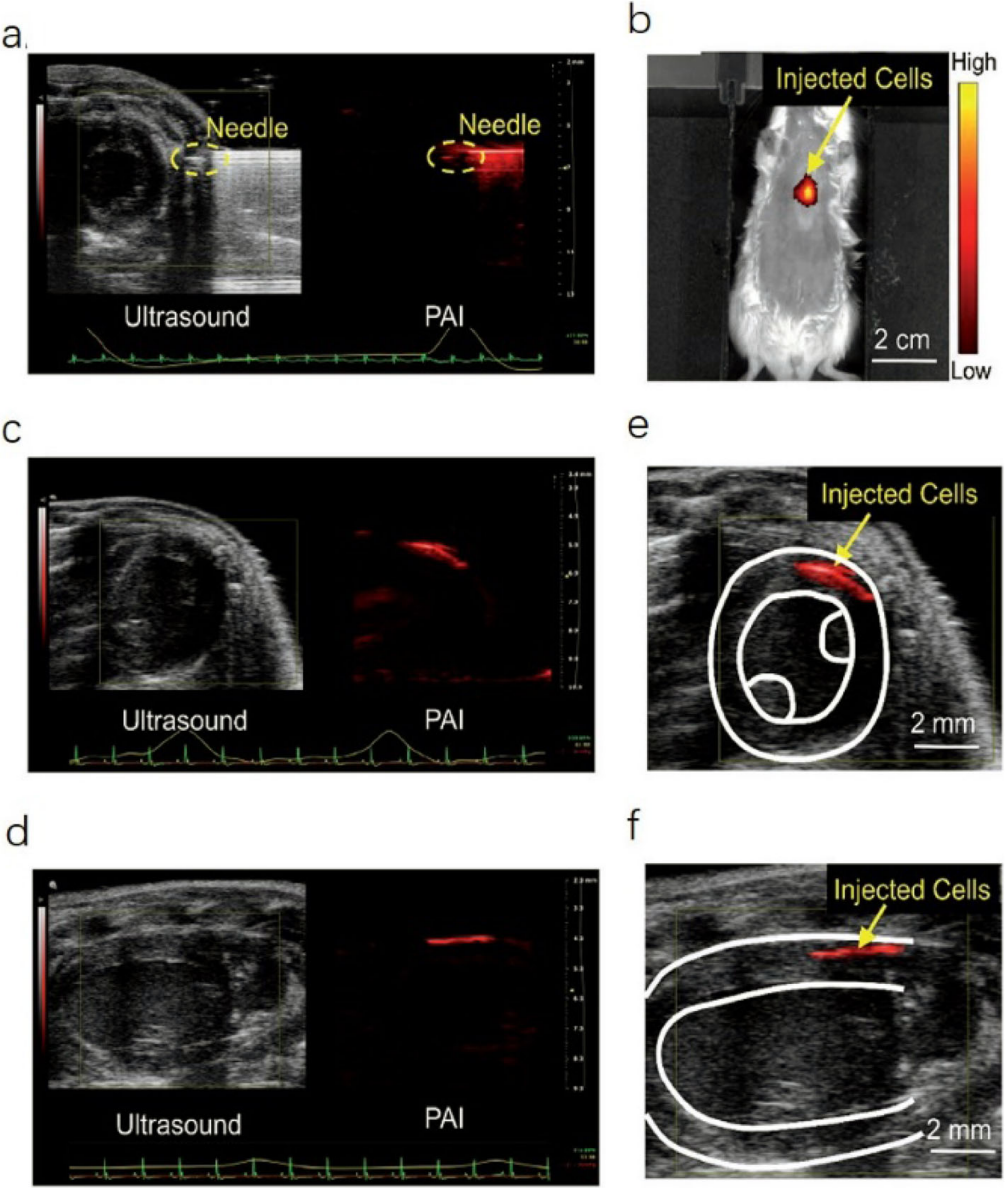
PAI of the injection and engraftment of PANP-labeled hESC-CMs in mouse hearts in vivo [17]

Photoacoustic imaging (PAI) is a new imaging method that combines the high contrast of optical imaging with the spatial resolution and penetration depth of ultrasound (US). The basic principle of this imaging can be simply summarized as imaging by detecting broadband ultrasonic waves excited by pulsed light. A pulsed laser (usually a nanosecond pulse) irradiates a sample, which absorbs the laser energy that is further translated into heat energy, leading to transient thermal expansion of the sample and a high-frequency mechanical pressure wave (i.e., ultrasonic). The US wave travels through the medium of transmission to the surface, such as in an US transducer, which is processed by a computer, and a photoacoustic (PA) image is obtained [1]. In addition, the similarity between PA and ultrasonic imaging methods enables the integration of the two modes, and there is a good synergy that confers the ability to visualize morphology, function, and molecular characteristics. Based on recent developments, an updated PA/US imaging system has integrated all US modes (B-mode, Doppler mode, contrast mode, etc.) into PAI [2]. Usually, PAI alone cannot provide appropriate anatomical information for the effective localization of PAI signals in animals. Although the method of white light superposition is convenient, the repeated imaging of the same animal at different time points often leads to misinterpretation of the signal positioning because of the difficulty of repositioning the animal [3]. Moreover, PA/US can account for the limitations and deficiency of the single PA model to improve the accuracy of early detection of diseases [4]. Vevo LAZR-X (Fujifilm Visualsonics, Fig. 1), a commercial US and PA multi-mode imaging system, interprets PA signals regarding anatomical structure information [5]. The Vevo LAZR-X uses ultra-high-frequency electronic linear probes, with a maximum characteristic high-frequency US detector (> 20 MHz), where high-frequency technology is not only applied in the ultrasonic system, but also in the PA system [6]. High frequency leads to ultra-high anatomical resolution, and the high acquisition frequency of the system can complete instantaneous imaging to make real-time dynamic images smooth and clear, to obtain anatomical images, hemodynamic details, and accurate positioning [7–9]. PA/US multimodal imaging can be used to obtain anatomical and physiological information. This system has been used in various preclinical studies, such as tumor detection and staging, neuroscience research, reproductive development research, in vivo stem cell tracing, lymph nodes, and thrombosis [10–12]. In clinical application, PA/US has been used to acquire images of a vasculature mimicking phantom, a contrast-enhanced rat GI tract in vivo, and a human forearm in vivo. This developed system is the first PA/US system based on a conventional clinical US machine and provides conveniences such as handheld operation, an intuitive user interface, complete portability, and real-time imaging [13]. In 2015, Garcia-Uribe et al. [14] developed a dual-modality PA and US imaging system to noninvasively detect sentinel lymph nodes based on the accumulation of methylene blue dye. Ultimately, they aimed to guide percutaneous needle biopsies and provide a minimally invasive method for axillary staging of breast cancer. In 2014, Daoudi et al. [15] reported a system that is a first step toward an affordable portable combined US-PA imaging system. The system performance was tested in vitro and in vivo by imaging a human finger joint. In 2015, Sun et al. [16] reported that a Vevo LAZR Fujifilm Visualsonics (high-frequency small animal PA/US imaging system) was used to investigate whether prophylactic edaradone (a free radical scavenger used to treat ischemic stroke in Japan) could prevent infarction and cognitive impairment in transient cerebral hypoxic-ischemic models in mice. Blood oxygen saturation (StO_2_) and blood flow were detected in the cerebral cortex. A schematic diagram of the transient hypoxia-ischemia (tHI) model. MCA/ICA/ECA, middle, internal, and external carotid artery. RCCA, the right common carotid artery (Fig. 2a). Doppler flow imaging and 3D reconstruction showed reduced perfusion in the ipsilateral hemisphere and the reversal of blood flow in the right internal carotid artery (RICA) in the Circle of Willis after right carotid artery occlusion (RCCAO) (Fig. 2b). PAI showed that StO_2_ in the ipsilateral cortex was reduced from ~ 70% to ~ 61% upon RCCAO and plummeted to ~ 20% upon exposure to hypoxic air (Fig. 2c). After 30 min tHI, the ipsilateral hemisphere showed poorer recovery of StO_2_ than the contralateral cortex (~ 28% compared with ~ 39% in normoxia and ~ 53% vs ~ 70% under 100% oxygen) (Fig. 2d) (*n* > 3 times). This pattern suggests that a transient episode of HI may impair subsequent cortical oxygenation. In 2018, Qin et al. [17] demonstrated that a semiconductor polymer (SP) combined with the PA properties of nanoparticles can trace these cells in mice. This is achieved by virtue of two benefits of the photoacoustic properties of nanoparticles (PANPs). First, strong PA signals and specific spectral features of SPs allow PAI to sensitively detect and distinguish a small number of PANP-labeled cells (2000) from background tissues in vivo. Second, the PANPs show a high efficiency for human embryonic stem cell—cardio myocytes (hESC-CMs) labeling without adverse effects on cell structure, function, and gene expression. US imaging can assist in the transplantation of these cells and can be used to evaluate cardiac repair therapy with high-resolution photosonography. PAI and US imaging were also performed to visualize the engraftment of transplanted cells in vivo. Figure 3a shows an ultrasound image of cardiac structures in a short-axis view, in which the injecting needle is difficult to be differentiated from the ultrasound image due to its duplicated artifacts and similar intensity to the tissues. An electrocardiogram and respiratory coupling were used to acquire B-mode US images and multi-spectral PA images from short-axis (Fig. 3c) and long-axis views (Fig. 3e). Their merged images in Fig. 3d and f showed the 3D spatial relationship between the transplanted cells and the host myocardium at a high resolution (~ 100 μm). In addition, the PANP-labeled cells also emitted a near-infrared fluorescence upon laser excitation (peak at 820 nm), which facilitated FI to further confirm the cell transplantation in vivo (Fig. 3b).

## PAI in the second near-infrared window (the second near-infrared PA)

The advantages of the second near-infrared (NIR-II) PAI are as follows: large penetration depth, low background noise, high maximum allowable irradiation energy and less absorption and scattering by skin tissues, which is conducive to achieving high-resolution imaging of deep tissues [18]. However, melanin, hemoglobin, and other biological components absorb and scatter light in the first near-infrared (650–970 nm) window, resulting in background interference and spontaneous fluorescence, which can reduce the sensitivity, spatial resolution, and contrast of PAI. In contrast, PAI has less spontaneous fluorescence interference in the display of NIR-II (1200–2000 nm) window and no background signal. Because of the lower light absorption and scattering of endogenous biomolecules in this area, it has higher sensitivity and spatial resolution and deeper tissue penetration depth. Endogenous PAI contrast agents in NIR-II include fat, collagen, and other components [19]. An external PAI contrast agent in NIR-II can locally enhance tissue absorption performance, enhance the PA signal, and improve image contrast. Therefore, the use of an external contrast agent in NIR-II is an important condition for deep tissue PAI [20].

**Fig. 4:**
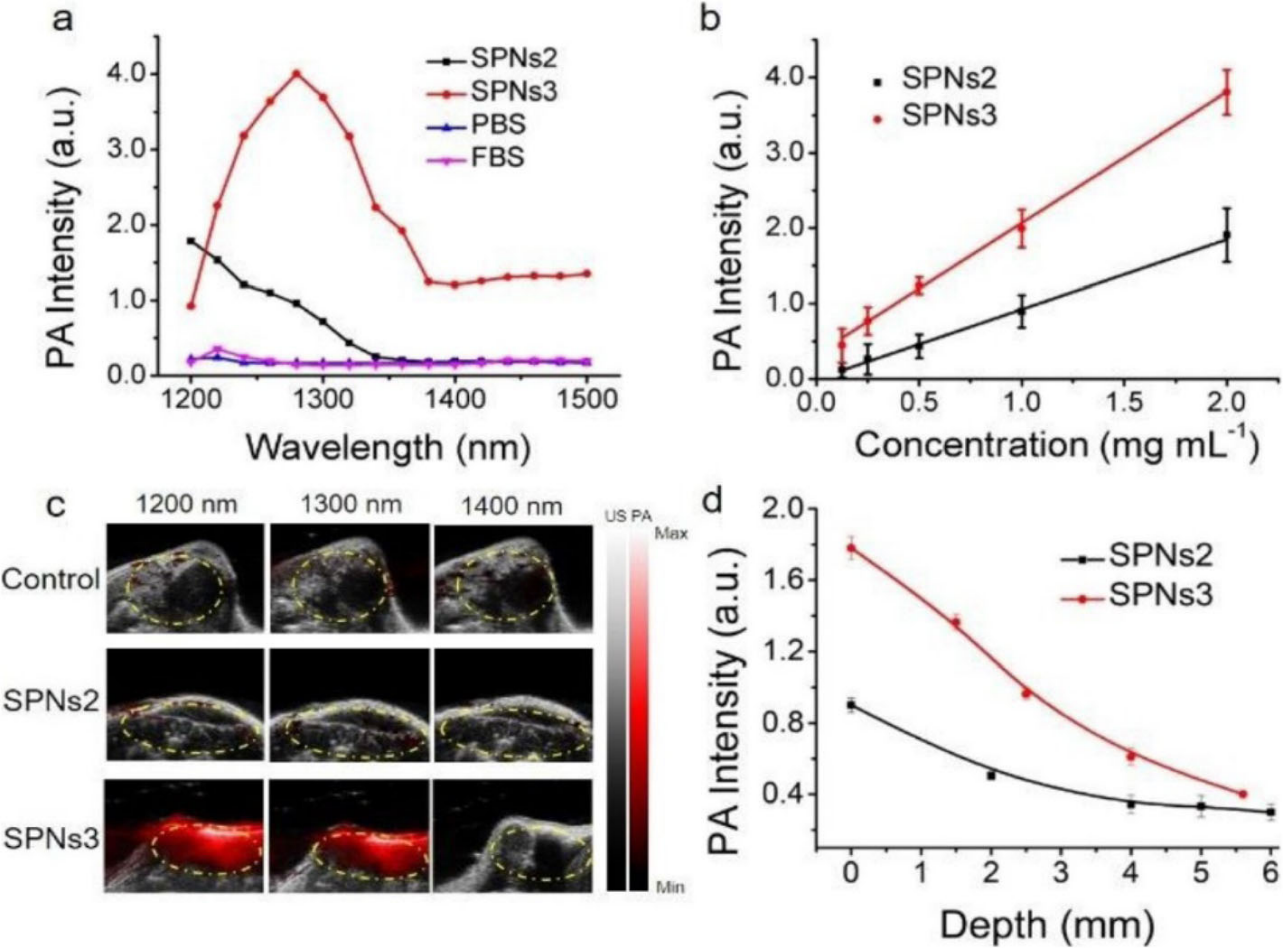
**a** PA spectra of 2 mg/mL SPNs2–3, PBS, and FBS; **b** PA signal of SPNs2–3 at various concentrations; **c** PA/US co-registered images without injection of SPNs2–3 (control) and with intratumoral injection of 50 μL, 500 μg/mL SPNs2–3 (dashed circles); **d** Fitted PA signal curves of SPNs2–3 solutions (1 mg/mL) at various depths in chicken breast tissue [22]

**Fig. 5:**
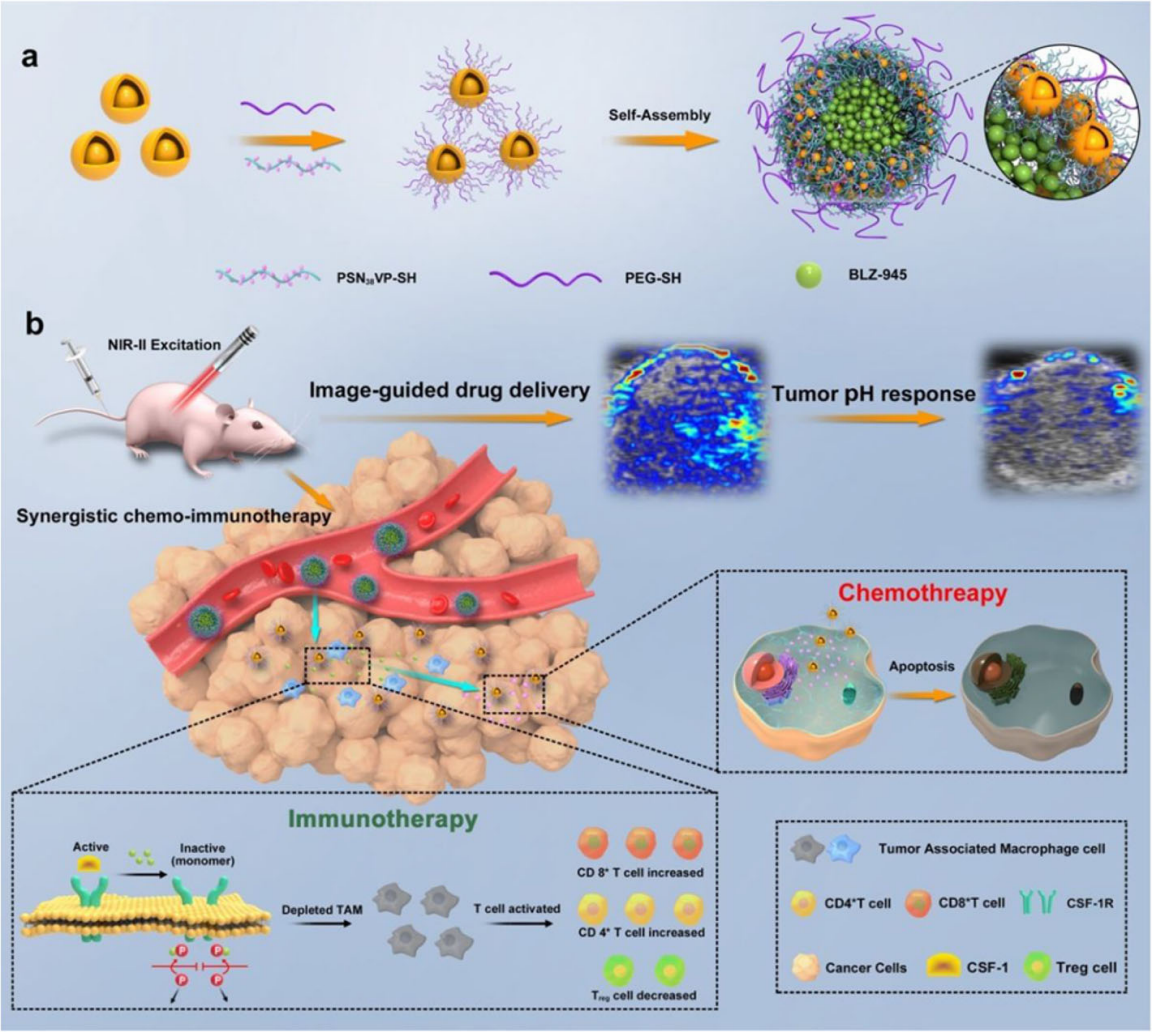
Preparation of dual-responsive AuNNP@SN_38_ Ve loaded with BLZ-945. The smaller AuNNP@PEG/PSN_38_VP then penetrated the deeper tumor regions and released the SN_38_ prodrug in the reductive environment, leading to tumor cell apoptosis. PA imaging in the NIR-II window further enabled drug release monitoring and guided therapy [23]

However, because of a lack of NIR-II absorbing contrast agents, PAI is most often carried out in the NIR-I window [21]. In 2019, Zhang et al. [22] reported semiconducting polymer nanoparticles (SPNs) composed of DPP-based two-acceptor semiconducting polymers with strong electron-deficient acceptors (Benzobisthiadiazole) to develop an efficient NIR-II PAI/photothermal therapy (PTT) agent. With high photothermal conversion efficiency (60%), SPNs3 exhibited strong PA signals at 1280 nm that were detected by commercial NIR-II PAI systems (Vevo LAZR-X), and PAI-imaging guided photothermal tumor therapy was realized in live animals under NIR-II light excitation. The PA intensity generated by SPNs3 at 1280 nm was 2.2-fold stronger than that of SPNs2 at 1200 nm. The PA signal of SPNs3 was 23 ~ 27-fold higher than that of FBS and PBS at 1280 nm, which could indicate an excellent PA signal-to-noise ratio in vivo. As depicted in Fig. 4a, the maximum PA signals of SPNs2–3 were observed at approximately 1200 and 1280 nm in the NIR-II PA spectra. In Fig. 4b, SPNs2–3 exhibit a good linear relationship between the PA signal intensity and concentration. After intratumoral injection of SPNs2–3 (Fig. 4c), from Fig. 4d, we found that the NIR-II PA signals of the SPNs3 solution were much higher than those of SPNs2 under chicken tissue of different thickness, exhibiting the good deep-tissue PA imaging capabilities of SPNs3. relatively stronger PA signals for SPNs3 were detected in the tumor region compared with those in the untreated group and the SPNs2-treated group, implying that SPNs3 is an available PA agent in the NIR-II window. In 2020, Zhu et al. [23] developed dual biologically responsive nanogapped gold nanoparticle (Au NPs) vesicles loaded with immune inhibitors and carried anticancer polymeric prodrugs for synergistic concurrent chemoimmunotherapy against primary and metastatic tumors, along with guided cargo release by PAI in the NIR-II window (Fig. 5).

## PTT

**Fig. 6:**
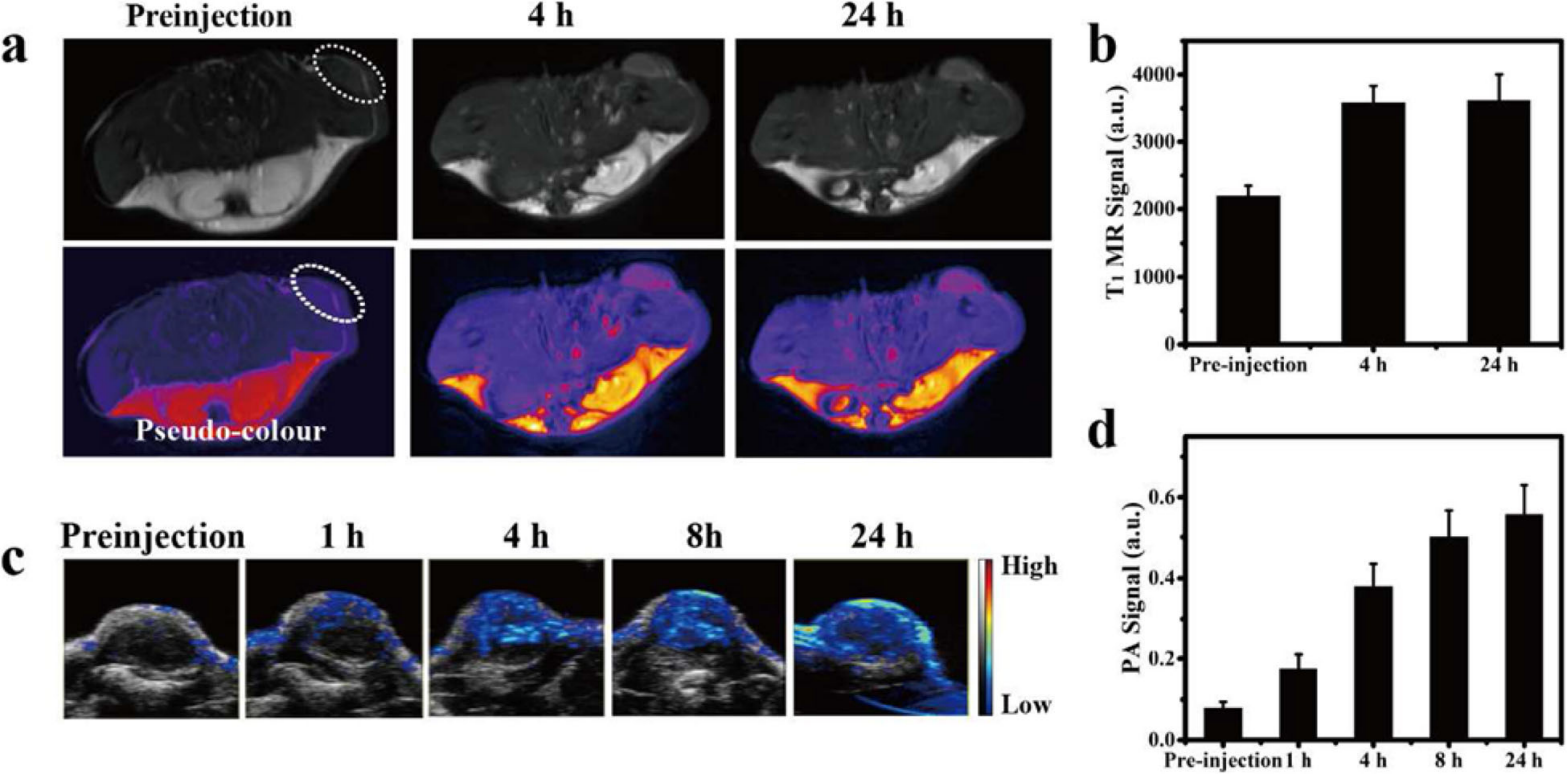
MRI and PAI performance in vivo [25]

**Fig. 7:**
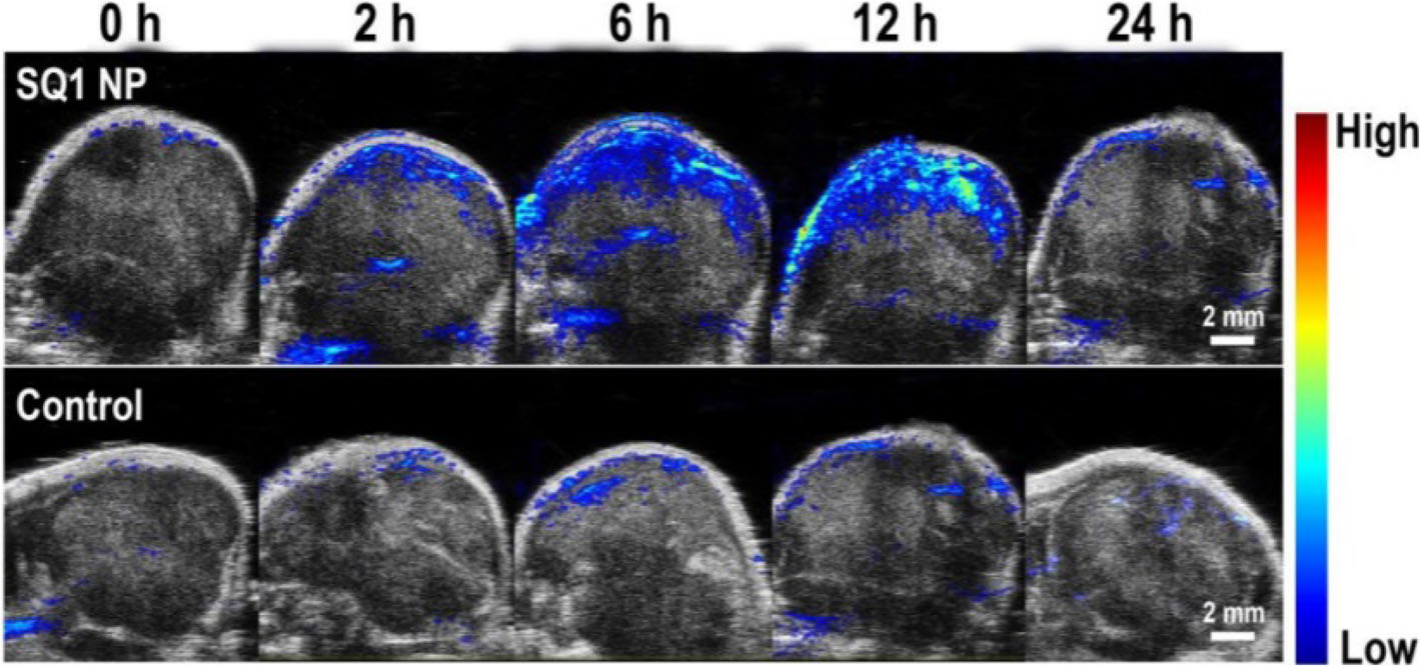
PAI of fibronectin-positive MDAMB-231 tumors at 0, 1, 2, 6, 12 and 24 h postinjection of SQ1 nanoprobe or SQ1@DSPE (control) i.v [26].

In recent years, PTT, which is a minimally invasive tumor treatment technique, has been developed. This treatment method uses a laser (mostly near-infrared laser) as the external energy source, absorbs near-infrared light with special photothermal therapeutic agent, converts the absorbed light energy into heat, causes the temperature of the tumor site to rise, and thus induces cell apoptosis or produces a direct lethal effect on cells. Because near-infrared lasers can penetrate the skin and tissue, effective thermal destruction of deep tumor tissue can be achieved without damaging normal cells. It has been widely used in accurate ablation of tumors with low systemic toxicity. In addition, PAI with high resolution and deep tissue penetration characteristics uses a naturally induced ultrasonic signal based on the photothermal effect of PTT reagent under pulsed near-infrared laser irradiation. The PA signal is primarily determined by photothermal conversion, which is the inherent principle of PTT, making PAI and PTT an ideal combination. PAI and PTT provide useful tools for diagnosis and treatment of diseases. However, current applications of PAI and PTT are greatly limited by their reliance on light sources in the first NIR-I window (750–1000 nm), which demonstrates shallow tissue penetration depth. This cannot satisfy the requirements of PAI and PTT conversion. NIR-II exogenous PAI contrast agent also exhibits good application prospects for PTT because of its strong absorbance and high conversion efficiency of photothermal spectral analysis [24]. It can greatly reduce the potential damage to normal tissue during PTT. In 2020, Liu et al. [25] reported an innovative Na_x_MnWO_3_-PEG nanoplatform constructed with protected cathodic Mn^2+^ for stabilized magnetic resonance imaging (MRI) contrast, demonstrating the ability to guide tumor PTT. The in vitro and in vivo results indicated that Na_x_MnWO_3_-PEG nanorods could be reliable nanoplatforms for MRI-guided PTT. Moreover, the changed valence of the tungsten element under redox status may contribute to the depletion of intracellular glutathione for further enhanced PTT. We believe that this brand-new concept will offer opportunities for developing more reliable and low-toxicity nanoagents for stable MRI, as well as more specific tumor therapy. In vivo T1-weighted MR images (Fig. 6a) and signals (Fig. 6b) of 4 T1-tumor-bearing mice before and after intravenously (i.v.) injection of NaxMnWO3-PEG nanorods (40 mg/kg). As shown in Figs. 6c and d, the PA signal could be detected as early as 1 h after i.v. injection of NaxMnWO3-PEG (40 mg/kg), and the signal intensity increased gradually over time and remained high even after 24 h, which was in accordance with the performance in MRI. Not only is PTT effective because it can lead to hyperthermia, it can also achieve photothermal ablation (PTA). Hyperpyrexia refers to the rise of tissue temperature to 42–46 °C, while irreversible PTA can lead to tissue necrosis, which is equivalent to 240 min at 43 °C or 1 s at 54 °C. Yao et al. [26] reported leveraging the excellent photothermal conversion capability under NIR irradiation to show a squaraine dye (SQ1) nanoprobe that performed well in both PAI and PTT of solid tumors. These results indicate that SQ1 nanoprobes have significant tumor targeting imaging capabilities. Moreover, a SQ1 nanoprobe can be used for PTA of tumors. Depending on the photothermal effect of the SQ1 nanoprobe, PAI can be carried out simultaneously (Fig. 7). The PAI signals in the tumor regions increased and reached the maximum value at 12 h.

## Photodynamic therapy

**Fig. 8:**
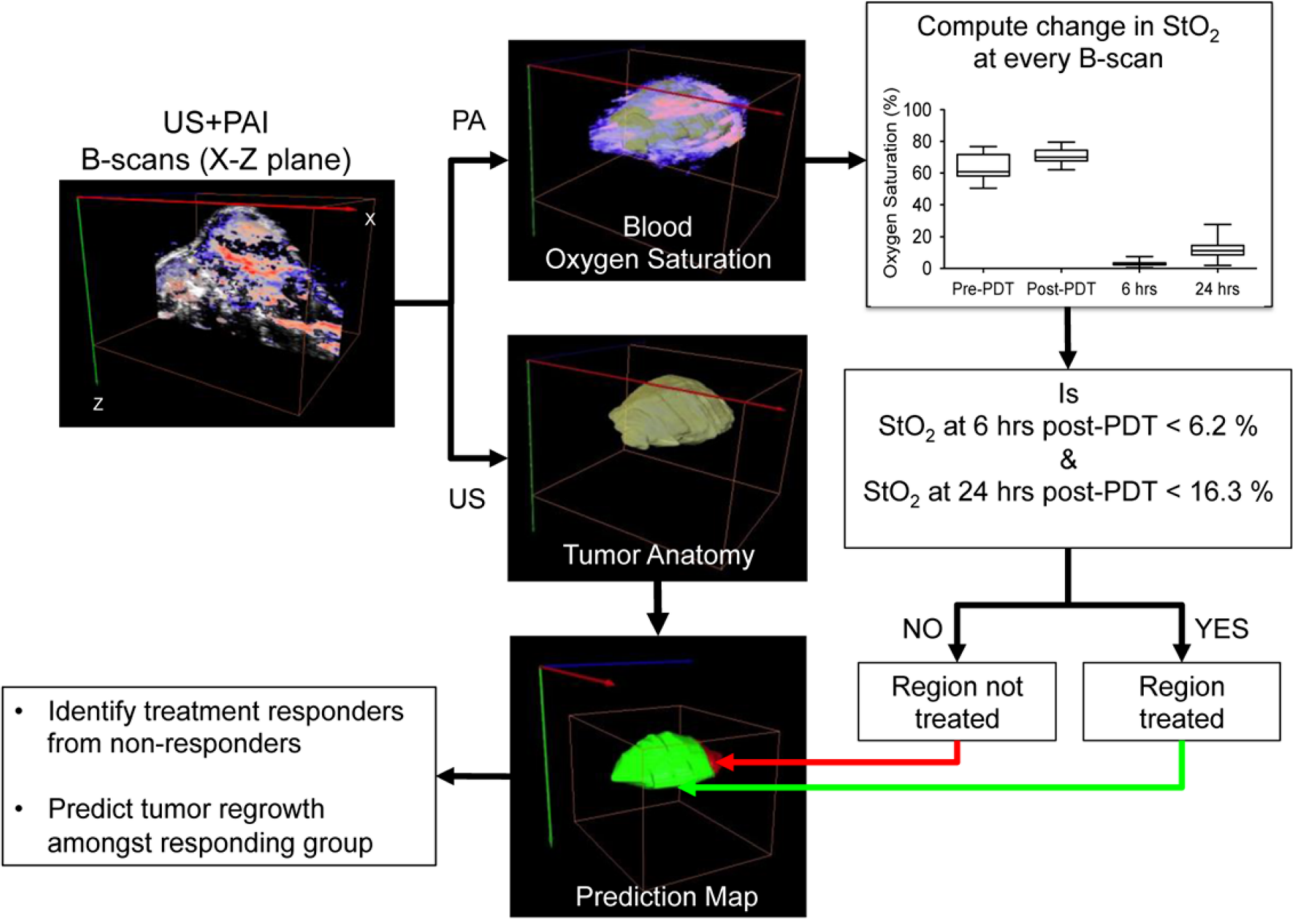
Schematic algorithm to obtain PDT treatment prediction map from the US and PA images for identifying treatment responders from non-responders and for prediction of tumor regrowth [28]

**Fig. 9:**
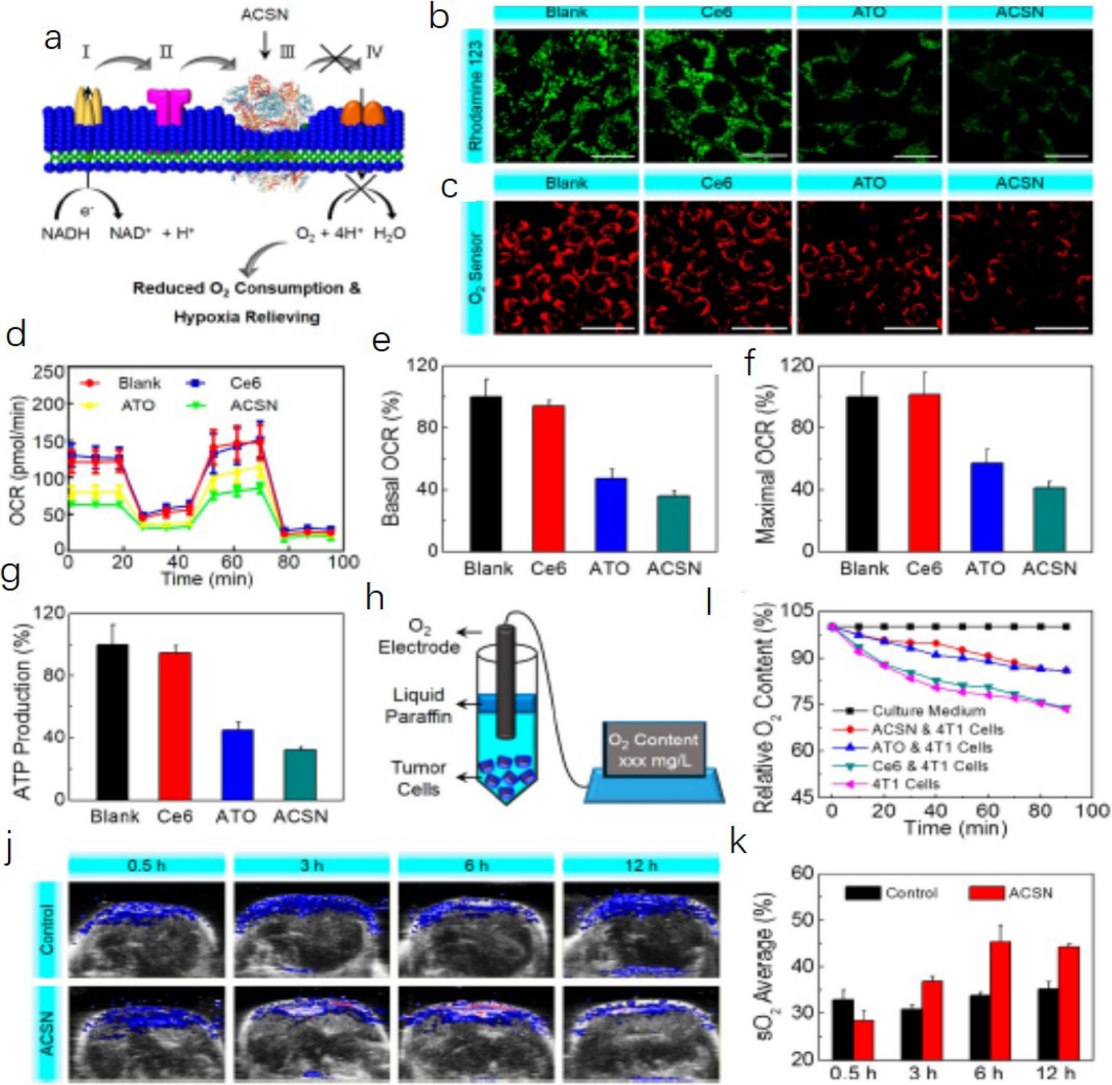
ACSN was injected i.v. to detect 4 T1 tumor-bearing mice by PAI [29]

Photodynamic therapy (PDT) is a photochemical-based, clinically used technique that produces cytotoxic substances by consuming oxygen, leading to cell death and vessel closure. Vascular damage reduces StO_2_. This causes a change in blood StO_2_. PDT as a therapeutic approach, and PAI as a noninvasive, reactive, and disease recurrence monitoring means, are a step toward this goal. Here, the author discussed that StO_2_ changes measured by PAI after PDT can be used as a detection method to predict the therapeutic effect and tumor recurrence [27]. In 2015, Mallidi et al. [28] investigated whether this change in StO_2_ measured by PAI post-PDT could act as a surrogate marker for predicting treatment efficacy and tumor recurrence. The findings of this study could possibly be used to guide and monitor several treatment modalities, such as PDT, radiation, and anti-angiogenic therapy, that involve a change in StO_2_. The author devised an algorithm to analyze the StO_2_ images of recurrent tumors obtained at various time points post-therapy. First, US and PAI B-scans were acquired at step sizes of 0.152 mm to obtain 3D maps of the anatomy and StO_2_. At every B-scan, the tumor region was mapped using US imaging and the average StO_2_ in the region was calculated. If the average StO_2_ at 6 h post-PDT and 24 h post-PDT in a particular B-scan frame was less than 6.2% and 16.3%, respectively, the B-scan region was considered treated and pseudo-colored as green; otherwise, the regions were pseudo-colored red to indicate no treatment (Fig. 8). Tumor hypoxia is the Achilles heel of oxygen-dependent PDT, and tremendous efforts are required to reverse tumor hypoxia. Zhao et al. [29] confirmed that a photosensitizer of chlorine e6 (Ce6)-based self-delivery nanomedicine (ACSN) effectively suppressed oxygen consumption to reverse tumor hypoxia by inhibiting mitochondrial respiration. Benefiting from the synergistic mechanism, an enhanced PDT effect for ACSN was observed regarding the inhibition of tumor growth. This self-delivery system for oxygen-economized PDT could be an appealing clinical strategy for tumor eradication. To verify whether this reduced O_2_ consumption could relieve tumor hypoxia, 4 T1-tumor-bearing mice were i.v. injected with ACSN for PAI detection. As depicted in Fig. 9a-i, schematic illustration of the proposed mechanism of ACSN for hypoxia remission by mitochondrial complex III inhibition. CLSM images of 4 T1 cells after being treated with Ce6, ATO, or ACSN and then stained by rhodamine 123. CLSM images of 4 T1 cells after being treated with Ce6, ATO, or ACSN in hypoxia and then stained by a Hypoxia/Oxidative Stress Detection kit. Seahorse XF24 Flux analysis of 4 T1 cells after treatment with Ce6, ATO, or ACSN for 12 h. The basal oxygen consumption rate (OCR), maximal OCR, and ATP production of 4 T1 cells after various treatments. Schematic illustration of the instrument to measure the O_2_ consumption of tumor cells. The dissolved O_2_ in a culture medium of 4 T1 cells after various treatments. As shown in Fig. 9j, the PA signals gradually increased over time, reflecting the increasing O_2_ content in the tumors. In particular, the signals peaked within 6 h and then decreased slightly. The average StO_2_ was also quantified, as demonstrated in Fig. 9 k.

## Multi-mode imaging

Multi-mode imaging is an important development in PAI that integrates a variety of imaging technologies and can provide a variety of types of information about the imaging region to improve the reliability, diversity, and authenticity of scientific research data. The study of PA multimode imaging can be generally divided into two aspects: PA/US imaging has inherent multi-parameter characteristics, which can be used to obtain multi-parameter PA/US images.

**Fig. 10:**
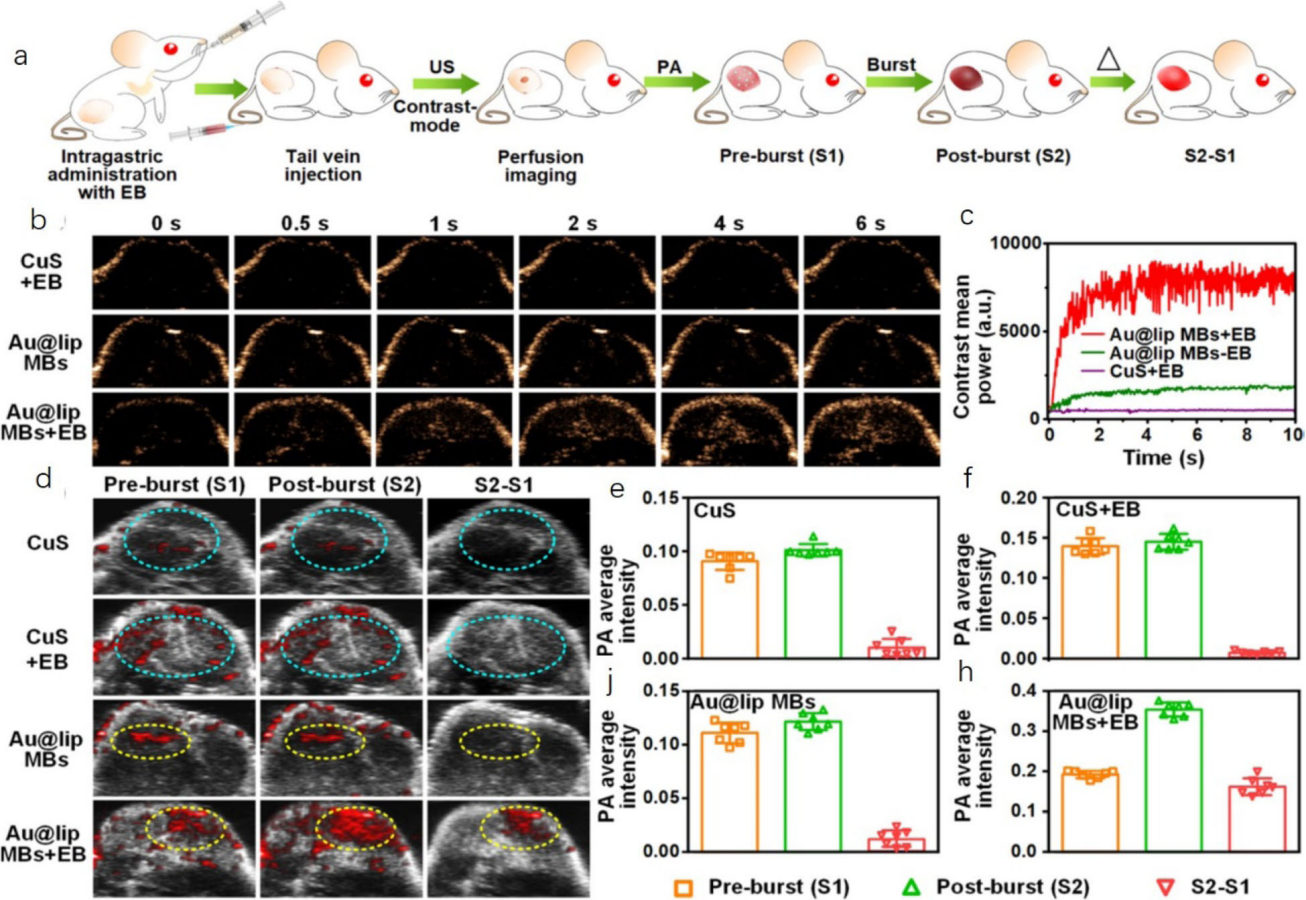
Background-free PAI to study tumor blood perfusion with Au@lip MBs [32]

Examples include PA images of blood StO_2_, total hemoglobin, deoxygenated hemoglobin, hypoxic hemoglobin, melanin, lipids, and other specific components. In addition, a variety of imaging parameters, such as tissue structure, blood flow velocity, and vascular perfusion, can be extracted by combining PA with ultrasonic signals [30, 31]. In 2019, Meng et al. [32] designed a US-responsive PA imaging probe based on microbubbles (MBs) containing Au NPs for in vivo “background-free” PA imaging. The obtained Au@lip MBs with separated Au NPs decorated within the lipid shell of MBs show low PA signals under near-infrared (NIR) excitation. Interestingly, under exposure to US pulses, these Au@lip MBs burst to form nanoscale aggregates of Au@lip NPs, which exhibit significantly enhanced NIR PA signals because of their red-shifted surface plasmon resonance. Scheme showing the experimental process. First, mice bearing CT26 tumors were orally treated with Erlotinib (EB) and then i.v. injected with Au@lip MBs for ultrasound imaging and photoacoustic imaging in sequence. Au@lip MBs were used as the contrast agents for both ultrasound and photoacoustic imaging. MB-based US imaging is an established method for studying blood perfusion in selected organs. Different from untreated tumors, in which US signals were detected only in their surrounding regions, strong US signals emerged in tumors immediately after the injection of Au@lip MBs and were dispersed throughout the tumor for mice with EB treatment (Fig. 10 a-c).

**Fig. 11:**
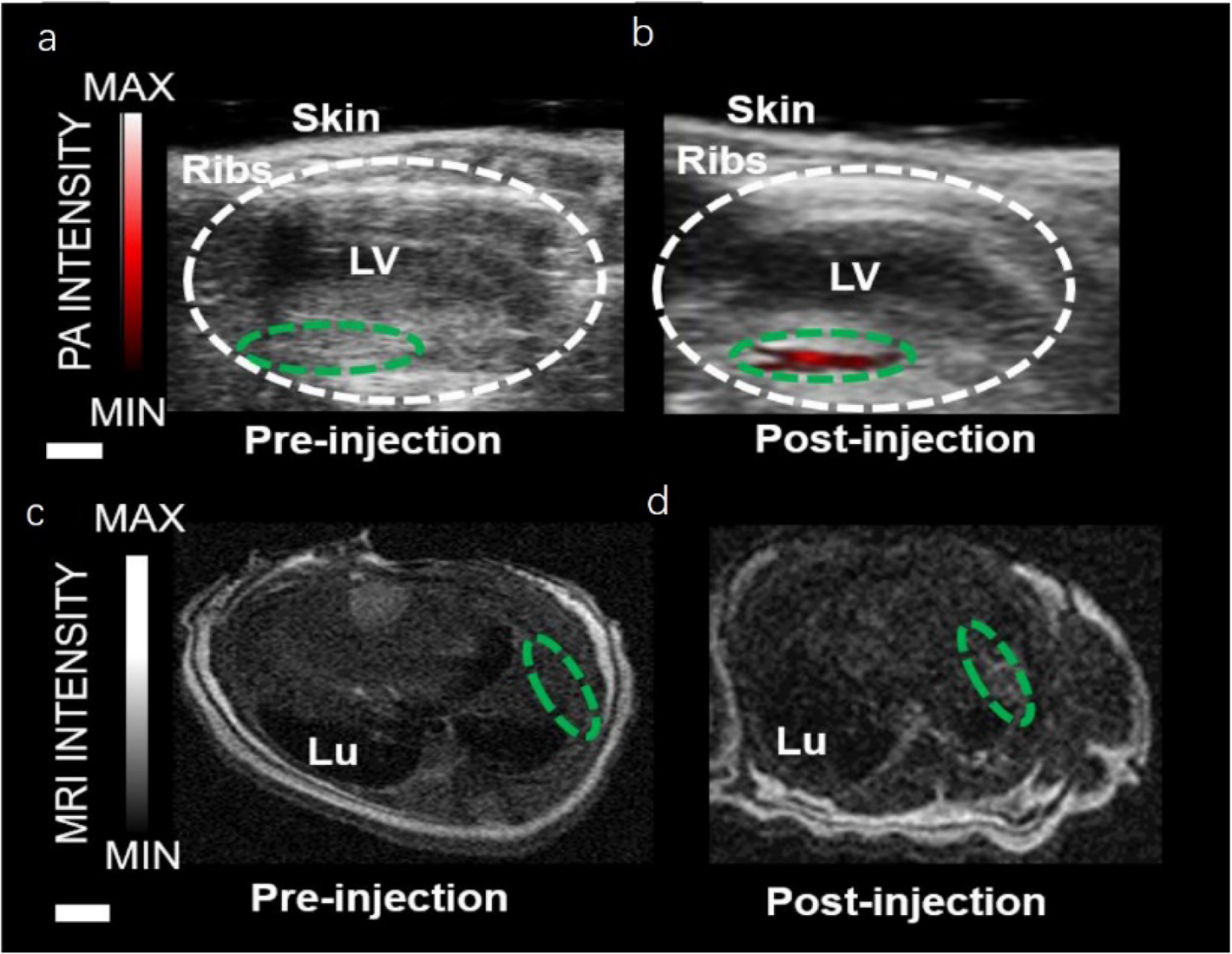
PAI and MRI of Gd (III)-SMNP implanted into mouse hearts [34]

In particular, for the tumor image in the untreated group with Au@lip MB injection, a large blood vessel happened to show up in the original PA image. By background subtraction, signals from this blood vessel could be completely eliminated to avoid false imaging results owing to the background interference (Fig. 10 d and j). Compared to tumors on untreated mice, those on mice after erlotinib treatment showed a significantly increased accumulation of Au@lip MBs (Fig. 10 d and h). In contrast, when the conventional probe CuS NPs were used, it became difficult to identify whether the observed PA signals in the tumor were from the NPs or just the background from tumor blood vessels (Fig. 10 d − f). The PA model was well combined with the US microvesicle imaging model to obtain the PA signal data of tumor vascular perfusion. Conversely, multi-mode contrast agents can be applied to a variety of imaging systems, such as PAI, nuclear magnetic imaging, computed tomography, optical imaging, and ultrasonic imaging, and have been used to improve the accuracy of scientific research diagnosis [33]. Lemaster et al. [34] in a 2019 study provided details of a synthetic melanin-based contrast agent for PAI and MRI. The most important finding is that the PA intensity increased dramatically upon incorporation of metal ions into polydopamine-based nanoparticles. Chelation is known to increase the biocompatibility of Gd-based contrast agents, which are clinically used in MRI. We used the Gd (III)-enhanced PA signal to image stem cells in vivo by coupling this modality with MRI. The labeled stem cells still expressed stem cell surface markers and continued to proliferate. In vivo experiments using 500,000 cells labeled with gadolinium-loaded synthetic melanin nanoparticles [Gd (III)-SMNP] particles were performed in mice (Fig. 11). Bone marrow mononuclear cells have been found to promote heart function and neovascularization after myocardial infarction via intramyocardial injection delivery. Echocardiograms pre- and immediately post-injection are also shown in Fig. 11, which indicate that the PA signal increased 64-fold.

## Summary and outlook

PAI technology combines the advantages of optical imaging and acoustic imaging, making the detection deeper, more accurate, and safer [35].

PAI employs non-ionizing laser radiation, which has higher biological safety than conventional CT imaging, MRI imaging, and other types of ionizing radiation. Therefore, it has become one of the most rapidly developing new biomedical imaging technologies. In particular, the multimode imaging system that integrates photoacoustic and ultrasonic imaging has emerged in recent years, as PAI and ultrasonic imaging demonstrate natural complementarity, and the system has a high degree of compatibility. The multimode real-time imaging system is realized by the organic integration of the two, to obtain imaging information and better meet the needs of scientific research for accurate detection and early diagnosis. The emergence of NIR-II PAI (1000 nm–2000 nm) has overcome the background interference and spontaneous fluorescence associated with NIR-I (650 nm–900 nm), thus improving the sensitivity, spatial resolution, and deeper tissue penetration depth of PAI.

Because of the inherent characteristics of PPT and PDT, PAI is the most effective detection method. PPT and PDT demonstrate good biological compatibility to avoid potential toxicity caused by their retention in the body, and high conversion efficiency to avoid damage to normal tissues. Therefore, they show good application prospects in present research. Multi-mode imaging and real-time imaging systems have become the focus of PA research and an important development trend for the future. In particular, the combination of PA and ultrasonic imaging can be used as an acoustic signal detection system. Moreover, the introduction of high-frequency technology into US and PAI can advance basic research. Meanwhile, multi-mode real-time imaging systems integrated with PA/US technology can provide more information, improve the accuracy of results, and better meet the needs of preclinical research for early and accurate diagnosis.

## Data Availability

All data analysed during this study are included in this published article.

## Abbreviations

PA: Photoacoustic
US: Ultrasound
PA/US: Photoacoustic/ultrasound
PANPs: Photoacoustic properties of nanoparticles
hESC-CM: Human embryonic stem cell—cardio myocytes
SP: Semiconductor polymer
tHI: Transient hypoxia-ischemia
RICA: Right internal carotid artery
RCCAO: Right carotid artery occlusion
StO_2_: Oxygen saturation
PAI: Photoacoustic imaging
PTT: Photothermal therapy
NIR-II: The second near-infrared
SPNs: Semiconducting polymer nanoparticles
PTA: Photothermal ablation
PDT: Photodynamic therapy
MRI: Magnetic resonance imaging
SQ1: Squaraine nanoprobe
ACSN: A photosensitizer of chlorine e6 (Ce6)-based self-delivery nanomedicine
MBs: Microbubbles
Au NPs: Gold nanoparticles
Gd (III)-SMNP: Gadolinium-loaded synthetic melanin nanoparticles
OCR: Oxygen consumption rate
i.v.: Intravenously
